# Association Analysis Between Ischemic Stroke Risk Single Nucleotide Polymorphisms and Alzheimer’s Disease

**DOI:** 10.3390/bioengineering12080804

**Published:** 2025-07-26

**Authors:** Wei Dong, Wei Wang, Mingxuan Li

**Affiliations:** Cerebrovascular Disease Department, Neurological Disease Center, Beijing Anzhen Hospital, Capital Medical University, Beijing 100029, China

**Keywords:** Alzheimer’s disease, ischemic stroke, single nucleotide polymorphism, genetics, biomarker, neuroimage

## Abstract

Alzheimer’s disease (AD) and ischemic stroke (IS) are prevalent neurological disorders that frequently co-occur in the same individuals. Recent studies have demonstrated that AD and IS share several common risk factors and pathogenic elements, including an overlapping genomic architecture. However, the relationship between IS risk gene polymorphisms and AD has been less extensively studied. We aimed at determining whether IS risk gene polymorphisms were associated with the risk of AD and the severity of AD in AD patients. We utilized data of AD patients and normal controls (NCs) sourced from the Alzheimer’s Disease Neuroimaging Initiative (ADNI) cohort. IS risk single nucleotide polymorphisms (SNPs) were identified through the most recent and largest IS genome-wide association study (GWAS) meta-analysis. Subsequently, we conducted SNP-based association analysis of IS-risk SNPs with the risk of AD, along with amyloid, tau, and neuroimaging for AD. The generalized multifactor dimensionality reduction (GMDR) model was used to assess the interactions among IS-risk SNPs and *apolipoprotein E* (*ApoE)* ε4. Protein–protein interactions (PPIs) of the IS-risk genes product and APOE were explored using the STRING database. Seven IS-risk SNPs were involved in the study. Five SNPs were found to be associated with at least one measurement of cerebrospinal fluid (CSF) levels of amyloid-beta 1–42 (Aβ_42_), total tau (t-tau), and phosphorylated tau 181 (p-tau_181_), as well as the volumes of the hippocampus, whole brain, entorhinal cortex, and mid-temporal regions. After multiple testing corrections, we found that T allele of rs1487504 contributed to an increased risk of AD in non-*ApoE* ε4 carriers. The combination of rs1487504 and *ApoE* ε4 emerged as the optimal two-factor model, and its interaction was significantly related to the risk of AD. Additionally, C allele of rs880315 was significantly associated with elevated levels of CSF Aβ_42_ in AD patients, and A allele of rs10774625 was significantly related to a reduction in the volume of the entorhinal cortex in AD patients. This study found that IS risk SNPs were associated with both the risk of AD and AD major indicators in the ADNI cohort. These findings elucidated the role of IS in AD from a genetic perspective and provided an innovative approach to predict AD through IS-risk SNPs.

## 1. Introduction

AD is an age-related neurodegenerative disorder and the most prevalent form of dementia [1]. Cerebrovascular disease is recognized as the leading cause of mortality among middle-aged and elderly individuals, with IS being the predominant clinical type [2]. The principal pathological features of AD involve the abnormal aggregation of Aβ plaques, along with the presence of neurofibrillary tau tangles in brain tissue [3]. In IS, the occlusion of the cerebral artery results in a deficiency of oxygen and glucose supplied by the obstructed vessel. The neuropathological changes in AD and IS both result in pathophysiological alterations such as excitotoxicity, neuroinflammation, and oxidative stress [4].

Risk factors for IS, such as hypertension, diabetes, and smoking, are also indirect risk factors for AD [5,6,7,8]. For example, diabetes accelerated the progression from mild cognitive impairment (MCI) to AD within the first year following an MCI diagnosis [9]. Cigarette smoking contributed to reduced cortical thickness in regions that exhibited significant atrophy in the early stages of AD [10], while effective management of blood pressure can mitigate cognitive decline and potentially alter the progression of AD [11].

IS is considered a risk factor for AD by the American Stroke Association [12]. In a meta-analysis of seven cohort studies and two nested case–control studies, for strokes, the pooled effect size for AD risk was 1.59 (95% confidence interval of 1.25–2.02; *p* = 0.000) [13]. Compared with AD patients who suffered from IS, patients with AD alone had better cognitive performances in the delayed recall test, language function, and semantic fluency [14]. In the study of AD autopsy cases, the result indicated a significant association between the occurrence of watershed cortical infarcts and AD, with rates of 32.4% in AD compared to 2.5% in controls [15]. The use of anti-stroke medicine such as Aspirin reduced the risk and development of AD [16,17]. An incident stroke is not a rare event in the AD population. When compared to NC, the incidence rate of intracerebral hemorrhage (ICH) in patients with AD was significantly higher; however, the incidence rate for IS was similar between the subjects with and without AD [18,19].

The ε4 allele of the *ApoE* gene is a major late-onset genetic risk factor for AD [20]. GWAS, and their meta-analyses have provided new insights into the genetic factors of both AD [21] and IS [22]. They identified the genome-wide SNPs that were significantly linked to AD or IS. In our previous study, we analyzed the common genetic factors and pathways in AD and IS based on GWAS [23]. Recent studies have indicated overlapping genetic parameters between AD and IS. A meta-analysis incorporating data from the Alzheimer’s Disease International Genomics Project and the METASTROKE consortium, which focused on small-vessel-stroke GWAS, identified a significant region (*ATP5H/KCTD2/ICT1*) associated with both conditions (*p* = 1.8 × 10^−8^) [24]. Furthermore, another investigation analyzing two extensive GWAS datasets for AD and IS revealed 16 pleiotropic genes significantly linked to both diseases. Notably, several of these genes (*EPHA1*, *MS4A4A*, *UBE2L3*, and *TREM2*) were involved in immune system functioning [25]. However, to date, there have been no association studies examining the relationship between IS risk gene polymorphisms and the risk of AD.

In our study, the first objective was to determine whether IS risk SNPs were associated with the risk of AD. The second aim was to assess whether the interactions among IS risk SNPs and *ApoE* ε4 were connected to the risk of AD. Additionally, we discover whether IS risk gene products and APOE are interconnected and indirectly associated with the risk of AD. The third aim was to evaluate whether IS risk SNPs were related to major AD CSF biomarkers in AD patients. The last objective was to determine whether IS risk SNPs were related to the AD neuroimaging biomarkers in AD patients. These findings may clarify the role of IS in AD from a genetic perspective and offer a novel approach for predicting the onset and severity of AD through IS risk SNPs.

## 2. Materials and Methods

### 2.1. Participates

The data used in this research were obtained from the ADNI database. The initiative known as ADNI, which began as a collaboration between public and private sectors, was spearheaded by Principal Investigator Dr. Michael W. Weiner. The main aim of ADNI was to determine if the combined use of serial magnetic resonance imaging (MRI), positron emission tomography (PET), a range of biological markers, and clinical and neuropsychological evaluations could effectively assess the advancement of MCI and early AD. Approval for the ADNI protocol was granted by the institutional review boards of all involved institutions, and written informed consent was secured from all participants or their guardians. For further details, please visit www.adni-info.org (accessed on 15 October 2024). Our analysis focused specifically on data from the ADNI2 sub-groups, leading to a final cohort comprising 127 AD and 155 NC patients.

### 2.2. Genotyping Data

ADNI2 samples were genotyped using the HumanOmniExpress BeadChip (Illumina, San Diego, CA, USA) (730,525 markers). In our study, we selected the IS risk SNPs based on the latest and largest IS GWAS meta-analysis [22]. SNPs were filtered to ensure a minimum effect allele frequency (EAF) of 0.05 following the Hardy–Weinberg (H-W) equilibrium.

### 2.3. CSF Biomarker Measurements

Data regarding the levels of AD CSF biomarkers were also gathered from the ADNI database. All CSF samples were collected and then quickly frozen using dry ice, after which they were transported immediately to the ADNI Biomarker Core laboratory located at the University of Pennsylvania Medical Center. Subsequently, the samples were thawed at room temperature, gently mixed, and prepared into aliquots of 0.5 mL. Finally, the measurements of CSF Aβ_42_, t-tau, and p-tau_181_ utilized the multiplex xMAP Luminex platform (Luminex Corp, Austin, TX, USA) along with the INNOBIA AlzBio3 kit (Fujirebio, Ghent, Belgium).

### 2.4. Brain Structures on MRI

The MRI data acquisition protocol used in ADNI subjects has been described in https://adni.loni.usc.edu/data-samples/adni-data/neuroimaging/mri/ (accessed on 15 October 2024). In this study, hippocampus, entorhinal, whole brain, and mid-temporal volumes were defined as regions of interest.

### 2.5. Statistical Analysis

Statistical analyses were conducted utilizing SPSS software version 26.0 (SPSS Inc. in Chicago, IL, USA). First, we analyzed the baseline information. Unpaired t-test was used to evaluate the differences in age and the volumes of the hippocampus, entorhinal, whole-brain, and mid-temporal regions. To investigate group disparities in years of education and levels of CSF Aβ_42_, CSF t-tau, and CSF p-tau_181_, the Mann–Whitney U test was applied. The chi-squared test was employed to assess group differences in gender distribution and *ApoE* ε4 status. Subsequently, we examined the association between IS risk SNPs and the risk of AD. Logistic regression analysis, adjusting for factors such as age, gender, education level, and *ApoE* ε4 status, was used to compare allele frequencies of IS risk SNPs between AD patients and NC. Subsequently, the association between the A allele of rs1487504 and the risk of AD was examined by stratifying based on the *ApoE* ε4 status, adjusting for factors such as age, gender, and education levels. The assessment of interactions among IS risk gene polymorphisms and *ApoE* ε4 was conducted using GMDR software (version 0.9). Additionally, STRING software (https://string-db.org/, accessed on 15 May 2025) was employed to analyze PPI networks, facilitating the discovery of potential connections among IS risk gene expression products and ApoE. Finally, we assessed the relationships between IS risk SNPs and CSF, as well as neuroimaging biomarkers, in patients with AD. Correlations between IS risk genotypes and levels of CSF Aβ_42_, tau, and p-tau_181_, in addition to brain imaging data, were evaluated in AD cohorts through multiple linear regression models, correcting for age, gender, education years, and *ApoE* ε4 status. Multiple comparison correction was applied using Bonferroni correction. A difference with a *p*-value < 0.05 after Bonferroni correction (*p*-value < 0.00714 before Bonferroni correction) was considered to be statistically significant. As for the missing values, multiple imputation with chained equations was conducted using the mice package in R version 4.1.2 to mitigate potential bias, under the assumption that the data were missing at random [26]. Data visualization was executed with GraphPad Prism software (version 9.0.0).

## 3. Results

### 3.1. Baseline Information of Participants

The dataset comprised 282 individuals from the ADNI2 cohort, including 127 individuals diagnosed with AD and 155 individuals classified as NC. The demographic and clinical characteristics, neuropsychological assessments, imaging results, and AD biomarker information of the study participants are summarized in Table 1. Patients with AD exhibit an “older-appearing” brain, lower levels of CSF Aβ_42_, and higher levels of CSF, t-tau, and p-tau_181_.

### 3.2. Characteristic of Enrolled SNPs

In the ADNI2 study, we enrolled seven IS risk SNPs. These SNPs were recognized as risk alleles for acute IS, large artery stroke, and small vessel stroke. The minimum EAF observed was 0.101, and no deviation from the H-W equilibrium was detected (Table 2).

### 3.3. Association of IS-Risk SNPs with AD

The analysis revealed no significant association between IS risk loci and AD after Bonferroni correction (*p* < 0.00714). The smallest *p*-value prior to the Bonferroni correction was 0.038 for the variant rs1487504. The forest plot displayed the connection between IS risk SNP associations and the risk of AD (Table 3). Notably, the A allele of rs1487504 was found to significantly increase the risk of AD in individuals without the *ApoE* ε4 allele (*p* = 0.006, OR = 2.899, Table 4).

### 3.4. The Impact of SNP–SNP Interactions on AD Risk and PPI Analysis

In the SNP–SNP interaction analysis, the combination of rs1487504 and *ApoE* ε4 emerged as the optimal two-factor model, demonstrating a significant interaction characterized by a cross-validation consistency of 10 out of 10, a sample testing accuracy of 70.43%, and a statistically significant *p*-value of 0.001 (Table 5).

**Figure 1:**
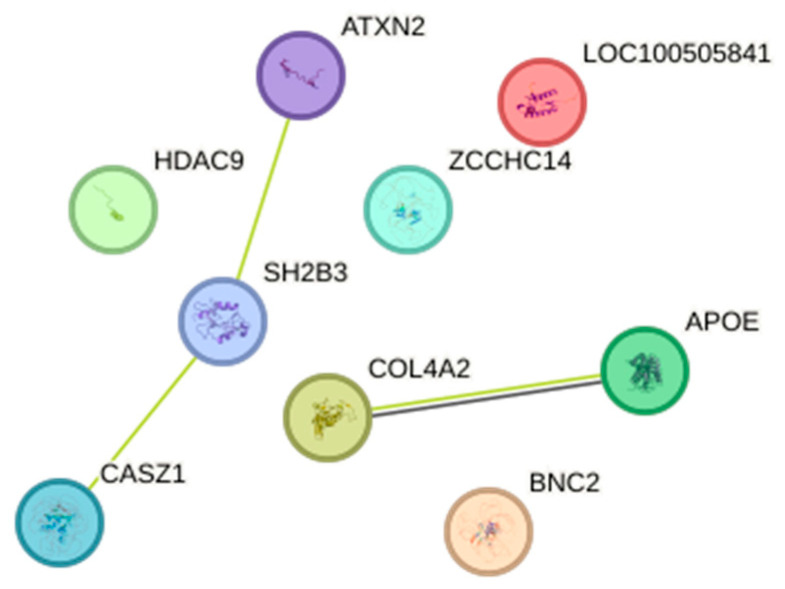
Protein–protein interaction network using STRING database.

Protein–protein interactions are illustrated in Figure 1. No interaction was studied between BNC2 and ApoE. SH2B3 and ATXN2, SH2B3 and CASZ1, and COL4A2 and ApoE were co-mentioned in PubMed abstracts. COL4A2 and *ApoE* were co-expression genes, but the total interaction score was only 0.455 (medium confidence).

### 3.5. Association of IS Risk SNPs with AD CSF Biomarkers

**Figure 2:**
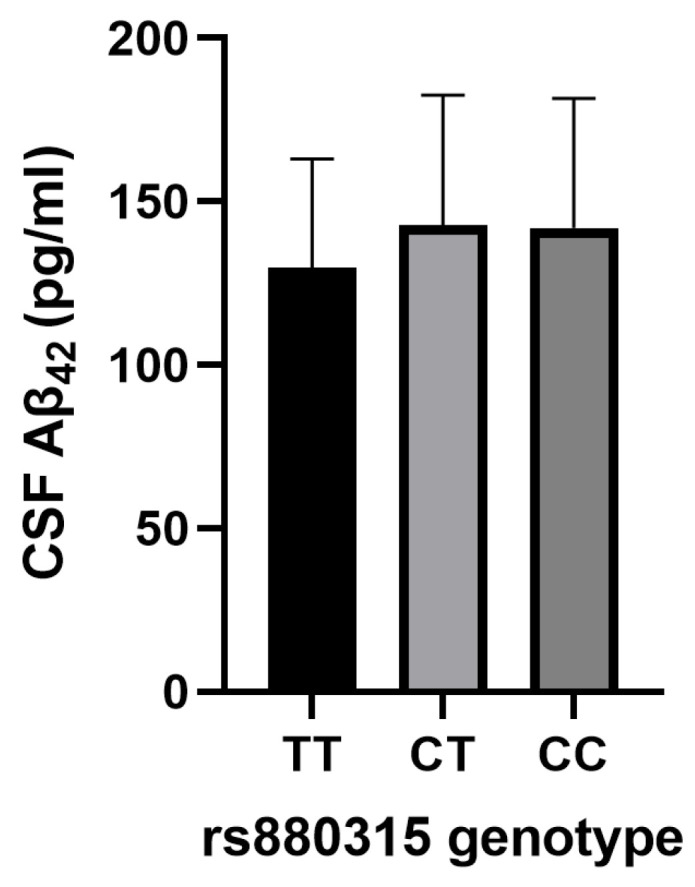
The distribution of CSF Aβ_42_ levels across different genotypes of SNP rs880315 in AD patients. Data are displayed as mean ± standard deviation. Aβ_42_, β-amyloid (1–42); AD, Alzheimer’s disease; CSF, cerebrospinal fluid; SNP, single-nucleotide polymorphism.

The A allele of rs1487504 was associated with decreased CSF Aβ_42_ levels (β = −0.197, *p* = 0.018, Table 6) and increased CSF t-tau levels (β = 0.177, *p* = 0.038, Table 6). Additionally, the A allele of rs17148926 was associated with an increase in CSF p-tau_181_ levels (β = 0.18, *p* = 0.039, Table 6). Furthermore, the C allele of rs880315 showed a significant association with increased CSF Aβ_42_ levels (β = 0.238, *p* = 0.004, Table 6 and Figure 2) after Bonferroni correction.

### 3.6. Association of IS-Risk SNPs with Neuroimaging Biomarkers in AD Patients

**Figure 3:**
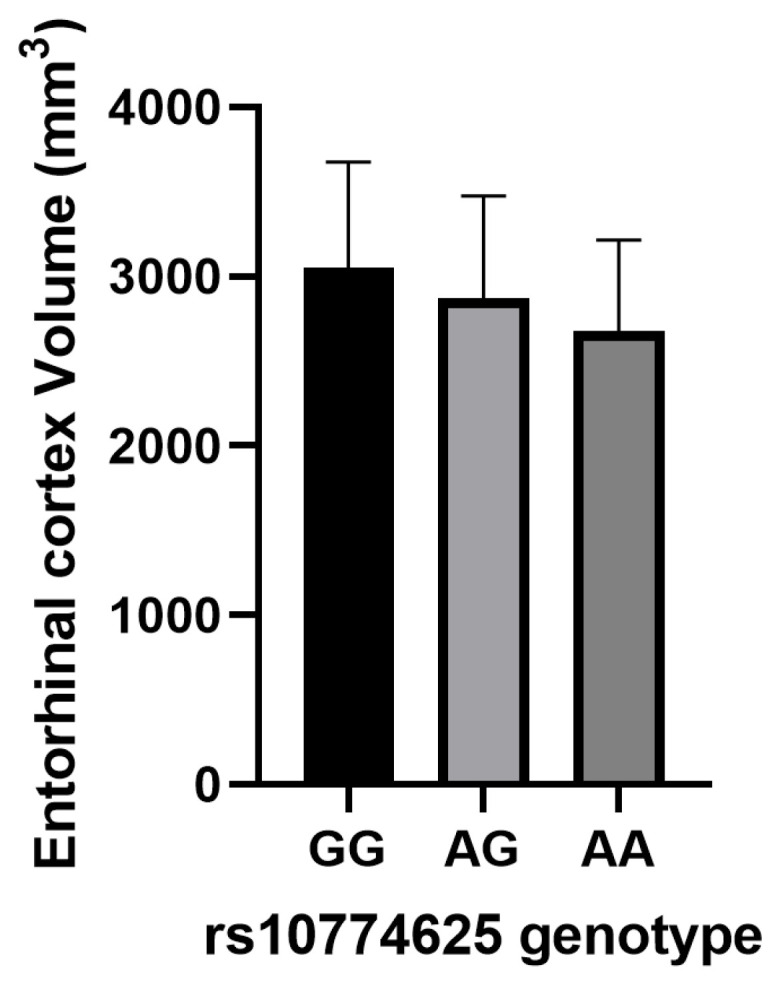
The distribution of Entorhinal cortex volume across different genotypes of SNP rs10774625 in AD patients. Data are displayed as mean ± standard deviation. AD, Alzheimer’s disease; SNP, single-nucleotide polymorphism.

The A allele of rs2107595 was associated with decreased volumes in the hippocampus (β = −0.178, *p* = 0.031, Table 7) and entorhinal cortex (β = −0.196, *p* = 0.026, Table 7). Similarly, the A allele of rs10774625 correlated with diminished whole brain volumes (β = −0.149, *p* = 0.049, Table 7) and was significantly associated with entorhinal cortex volumes (β = −0.249, *p* = 0.004, Table 7 and Figure 3) after Bonferroni correction. Conversely, the C allele of rs880315 was linked to increased volumes in the mid-temporal lobe (β = 0.167, *p* = 0.039, Table 7).

## 4. Discussion

Currently, no study has directly investigated the relationship between IS risk SNPs and AD. Our study mainly investigated the association of IS risk SNPs with the risk of AD among all participants, as well as AD-related CSF protein levels and MRI brain structures in AD participants. We discovered that the A allele of SNP rs1487504 was significantly associated with an increased risk of AD in *ApoE* ε4 non-carriers. In the SNP–SNP interaction study, the combination of rs1487504 and *ApoE* ε4 emerged as the optimal two-factor model, and its interaction was significantly related to the risk of AD. As for the PPI analysis, the gene products of IS-risk genes and APOE did not exhibit high confidence. Additionally, with respect to the rs10774625, rs880315, rs1487504, rs17148926, rs2107595, and AD-related CSF biomarkers, neuroimages showed trends towards associations, but these did not reach statistical significance. These findings suggest that most IS risk loci promote deterioration in AD, while rs880315 may serve as a potential protective factor.

We discovered that the A allele of SNP rs1487504 was significantly associated with an increased risk of AD in *ApoE* ε4 non-carriers. These indicated that rs1487504 may predict the risk of AD in *ApoE* ε4 non-carriers. *BNC2*, the nearest gene to rs1487504, is a zinc finger transcription factor recently identified as a core transcription factor essential for myofibroblastic activation in fibrosis, leading to extracellular matrix (ECM) deposition during fibrogenesis [27].

Currently, there are no relevant reports on the direct relationship between either BNC or rs1487504 and AD. BNC2 was a cardioprotection-related zinc finger biomarker during off-pump coronary artery bypass grafting [28]. BNC may be involved in heart failure (HF)-related pathogenic pathways and subsequently drive the activation or suppression of downstream signaling pathways during the progression of HF [29]. HF was linked to a heightened risk of AD in older adults [30,31]. In mouse models, chronic HF led to a diminished ejection fraction and was associated with increased permeability of the blood–brain barrier (BBB) and decreased cerebral blood flow (CBF). Additionally, depositions of Aβ were observed, supporting the link between HF and Aβ deposition [32,33]. In experimental studies conducted with rats, the cessation of blood flow prompted the expression of diffuse Aβ peptide and amyloid precursor protein within the hippocampus, entorhinal cortex, and neocortex [34]. HF did not lead to sudden neuronal death; rather, neurons experienced a metabolic energy crisis, excessive production of reactive oxygen species, impaired signal transduction, and neurotransmitter failure, with axonal microtubule disruption resulting in tau hyperphosphorylation [35]. The A allele of rs1487504 may contribute to decreased levels of BNC, thus increasing the risk of AD through HF.

Notably, the rs1487504 A allele was significantly associated with the risk of AD only in *ApoE* ε4 non-carriers. In the SNP–SNP interaction analysis, the combination of rs1487504 and *ApoE* ε4 emerged as the optimal two-factor model. However, no interaction was studied between BNC2 and ApoE in the PPI analysis. Individuals carrying one ε4 allele exhibit a 2–3 fold increase in the risk of AD, whereas those possessing two ε4 alleles demonstrate a 10–15 fold heightened risk [36,37]. In a cross-sectional convenience sample of 1610 participants, lower levels of Aβ_42_ and higher ratios of p-tau _181_/Aβ_42_ in CSF were observed among *ApoE* ε4 carriers compared to non-carriers in both the AD and NC groups. In a longitudinal cohort of 254 participants, the associations of CSF Aβ_42_ and p-Tau_181_/Aβ_42_ with cognitive decline were found to be stronger in *APOE* ε4 carriers than in non-carriers [38]. Another ADNI study also found that individuals carrying one or two *ApoE* ε4 alleles exhibited significantly elevated levels of t-tau and p-tau while showing reduced levels of Aβ_42_ in comparison to individuals without *ApoE* ε4 alleles [39].

In our study, the A allele of rs1487504 was associated with decreased CSF Aβ_42_ and increased CSF t-tau levels, which is similar to the effect of the *ApoE* ε4 allele. Our study also revealed a significant interaction between the *APOE* ε4 allele and rs1487504. AD patients carrying the A allele of rs1487504, who do not possess the *APOE* ε4 allele, exhibited a markedly higher risk of AD compared to other genotype combinations. This observation suggests a synergistic effect, in which the presence of both high-risk genotypes exacerbates the likelihood of suffering from AD. These findings underscore the critical role of genetic interactions in modulating AD outcomes, suggesting that *ApoE* ε4 may obscure the underlying connection between rs1487504 and AD risk, though no connections between *BNC2* and *APOE* were identified in the PPI network. Future studies should further investigate the combined effects of these genetic factors on AD pathogenesis.

The relationship between the A allele of rs1487504 and decreased CSF Aβ_42_ levels and increased CSF t-tau levels in AD patients showed trends towards associations but did not reach statistical significance. This indicated that rs1487504 may predict the development of worse outcomes in AD patients, but this needs further verification. Lipopolysaccharides (LPSs) were previously utilized to treat BV2 microglial cells, thereby inducing neuroinflammation. Functional experiments suggested that circ-Bnc2 could inhibit LPS-induced neuroinflammation in BV2 cells, leading to a reduction in HT22 cell apoptosis and an enhancement of proliferation [40]. Cognitive function was improved in the AD mouse model by mitigating the pro-inflammatory response of microglia and preserving their phagocytic and clearance capabilities regarding the deposited Aβ plaques [41]. The A allele of rs1487504 may contribute to decreased CSF Aβ_42_ and increased CSF t-tau levels in AD patients through neuroinflammation.

We discovered that the relationship between the A allele of rs17148926 and increased CSF p-tau_181_ levels showed trends towards associations but did not reach statistical significance. This suggested that rs17148926 may be a predictor of poorer outcomes in patients with AD. However, further validation is necessary. *LOX-ZNF474-LOC100505841* was found to be associated with cerebral white matter hyperintensities (WMHs) in the multi-trait analysis [42]. In AD patients, an increasing burden of hyperphosphorylated tau in the cortex independently predicted the severity of WMHs, which suggested that the presence of WMHs may indicate that tau cortical pathology is related to AD rather than being attributed to small vessel disease [43]. The A allele of rs17148926 in *LOC100505841* may be connected with enhanced p-tau deposition and further influence the cerebral WMH in AD.

rs880315 was associated with both CSF biomarkers and neuroimaging results. The rs880315 C allele was significantly correlated with higher CSF Aβ_42_ levels, and the linkage of the rs880315 C allele with larger mid-temporal lobe volumes exhibited trends towards associations but did not reach statistical significance. These indicated that rs880315 may predict the development of better outcomes in AD patients. At present, there is no study reporting on rs880315 in the context of AD. rs880315 is located in *CASZ1*, and its gene product CASZ1 acts as a novel regulator of T helper (Th) cell plasticity, with significant clinical implications for autoimmune inflammation and mucosal immunity. The absence of CASZ1 in CD4+ T cells reduced susceptibility to experimental autoimmune encephalomyelitis. The loss of CASZ1 during mucosal Candida infection significantly impaired Th17 and regulatory T cell (Treg) responses, consequently diminishing the mice’s ability to clear the secondary infection [44]. The single adoptive transfer of Aβ+ Tregs was sufficient to induce a reduction in Aβ accumulation and neuroinflammation associated with AD pathology in mice. Furthermore, Aβ-specific Tregs effectively inhibited inflammation in primary microglia induced by Aβ exposure [45]. Our study found that the rs880315 C allele was associated with elevated CSF Aβ_42_ levels and increased mid-temporal volume in AD patients. This suggests that the C allele of rs880315 may enhance the expression of *CASZ1* and potentially slow the progression of AD through T cell-associated inflammatory response.

The A allele of SNP rs10774625 was significantly linked to a smaller entorhinal cortex volume, and the association of rs10774625 with smaller whole brain volume showed trends towards associations but did not reach statistical significance. These suggest that rs10774625 may predict the development of worse outcomes in AD patients. At present, there is no report on rs10774625 in AD. rs10774625 is located within the intron of *ATXN2* but is linked to expression of *SH2B3* eQTL analysis [46]. rs10774625 showed nominal associations with systolic blood pressure [47] and Hashimoto’s thyroiditis [48]. Another ADNI study suggested that elevated blood pressure variability was associated with entorhinal cortex volume loss, particularly in ε4 carriers and individuals exhibiting AD biomarker abnormalities [49]. Abnormalities in the availability and/or metabolism of thyroid hormones (THs) have been hypothesized to contribute to AD and to serve as a risk factor for stroke. Recent findings confirmed that the TH derivative 3-iodothyronamine (T1AM) can restore synaptic function in the entorhinal cortex following transient ischemia, an effect that was also observed in an Aβ-enriched environment [50]. The A allele of rs10774625 may contribute to reduced entorhinal cortex and whole-brain volumes through mechanisms involving blood pressure variabilities and lower T1AM levels.

We discovered that the A allele of rs2107595, which is associated with smaller hippocampal and entorhinal cortex volumes, exhibited trends towards association; however, these trends did not reach statistical significance. These indicated that rs2107595 may serve as a predictor of poorer outcomes in AD patients. However, further validation is required to substantiate this finding. The rs2107595 A allele increased transcriptional capacity in luciferase assays and was correlated with elevated *HDAC9* mRNA levels in both primary macrophages and genome-edited Jurkat cells. In the ischemic reperfusion injury mouse model, the depletion of HDAC9 attenuated inflammation in the hippocampus, resulting in a reduced infarct volume and improved neurological function [51]. In AD, HDAC9-mediated CaM deacetylation induced hippocampus-dependent memory impairment [52]. Trichostatin A (TSA), a pan-inhibitor of HDAC9, facilitated the nuclear translocation of transcription factor EB. Administration of TSA to APP/PS1 mice increased the expression of autophagic and lysosomal genes in the brains of these mice, leading to improved memory. Accordingly, the burden of Aβ plaques was reduced [53]. HDAC9 was significantly downregulated in the prefrontal and visual cortices of AD subjects compared to controls [54]. Our study found that the rs2107595 A allele was associated with decreased volumes in the hippocampus and entorhinal cortex. These suggest that HDAC9 may promote neuroinflammation and increase Aβ burden in AD patients, with its expression varying across different cortical regions.

In our study, we discovered that the IS risk SNP rs1487504 may predict the risk of AD in *ApoE* ε4 non-carriers. The interaction of rs1487504 and *ApoE* ε4 was significantly related to the risk of AD. Additionally, SNPs rs10774625 and rs880315 may predict the development of AD, and rs17148926 and rs2107595 may be associated with the development trend of AD. The IS risk gene products and ApoE may not be closely connected with each other. Although it is widely accepted that the conversion of individuals to AD is influenced by multiple factors, this genetic approach aids in establishing causality, as genetic information remains unconfounded by environmental factors. Through reviewing published studies, we also identified potential mechanisms linking IS and AD associated with these SNPs (Table 8), which could serve as targets for maintaining optimal brain health threatened by AD or IS.

There are some limitations in our work. The limitation of our sample size affects statistical power when analyzing rare variants or stratifying data. The ADNI2 study focuses on the early detection and progression of AD, which may result in a participant pool that does not adequately represent the broader population of AD patients. Additionally, it does not explicitly include participants with IS, who could provide a more robust analysis of the overlap between IS and AD. Furthermore, the individuals within the ADNI cohort predominantly have European ancestry, which restricts the generalizability of the findings to other populations characterized by different ethnicities, genetic backgrounds, or environmental exposures. Further research involving larger clinical cohorts and more IS risk SNPs is necessary to validate the observed relationships. It is also essential to further elucidate the direct pathological connections and underlying mechanisms between IS risk gene products and AD.

## 5. Conclusions

In conclusion, this study investigated the potential relationship between IS risk SNPs and the risk and development of AD within the ADNI2 cohort. We identified rs1487504 as a novel genetic variant that may predict AD in non-*ApoE* ε4 carriers, as the interaction between rs1487504 and *ApoE* ε4 is significantly associated with the risk of AD. Four additional IS risk SNPs also demonstrated critical roles in major AD biomarkers and neuroimaging measures. These findings are significant for enhancing our understanding of the role of IS in AD from a genetic perspective and offer an innovative approach for predicting AD through IS risk SNPs.

## Figures and Tables

**Table 1:** The demographic, genetic characteristics, imaging, and CSF biomarker measures of the ADNI2 cohort.

	AD (N = 127)	NC (N = 155)	*p* Value
Age (years) ^1 2^	74.513 (8.39)	73.996 (6.019)	0.549
Gender (M/F) ^3^	75/52	80/75	0.212
Education (years) ^4 5^	16.0 (4.0)	16.0 (4.0)	0.052
*ApoE* ε4 (0/1/2) ^3^	23/59/45	116/35/4	<0.001
rs10774625 (AA/AG/GG) ^3^	21/70/36	36/72/47	0.573
rs12445022 (AA/AG/GG) ^3^	13/52/62	22/60/73	0.492
rs1487504 (AA/AG/GG) ^3^	2/28/97	2/21/132	0.085
rs17148926 (AA/AC/CC) ^3^	87/35/5	113/39/3	0.308
rs2107595 (AA/AG/GG) ^3^	3/49/75	4/43/108	0.105
rs880315 (CC/CT/TT) ^3^	17/51/59	13/66/76	0.344
rs9515201 (AA/AC/CC) ^3^	14/57/56	17/62/76	0.534
CSF Aβ_42_ (pg/mL) ^4 5^	129.0 (35.0)	205.0 (74.0)	<0.001
CSF t-tau (pg/mL) ^4 5^	117.0 (73.3)	57.1 (42.1)	<0.001
CSF p-tau_181_ (pg/mL) ^4 5^	48.6 (32.4)	28.3 (22.6)	<0.001
Hippocampus (mm^3^) ^1 2^	5992.07 (928.034)	7498.06 (860.923)	<0.001
Whole brain (mm^3^) ^1 2^	1,004,979.63 (113,512.084)	1,047,729.22 (103,976.995)	0.001
Entorhinal (mm^3^) ^1 2^	2890.39 (607.056)	3845.45 (622.302)	<0.001
Mid-temporal (mm^3^) ^1 2^	17,764.61 (3169.834)	20,571.16 (2293.72)	<0.001

Aβ_42_, β-amyloid (1–42); AD, Alzheimer’s disease; ADNI, Alzheimer’s Disease Neuroimaging Initiative; ApoE, apolipoprotein E; CSF, cerebrospinal fluid; M/F, male/female; NC, normal control; p-tau_181_, phosphorylated tau181; t-tau, total tau. The data are presented as the mean (standard deviation) ^1^ or median (interquartile range) ^4^; *p* values for continuous variables were from unpaired *t*-test ^2^ or Mann–Whitney U test ^5^; *p* value for categorical data was from the chi-square test ^3^.

**Table 2:** The characteristics of the enrolled IS-risk SNPs in ADNI2.

SNP	Main Phenotype	Gene	EA/OA	Function	EAF	H-W (*p*)
rs10774625	AIS	*SH2B3/ATXN2*	A/G	intron variant	0.454	0.792
rs12445022	SVS	*ZCCHC14*	A/G	intergenic variant	0.322	0.125
rs1487504	AIS	*BNC2*	A/G	intergenic variant	0.101	0.463
rs17148926	AIS	*LOC100505841*	A/C	intron variant	0.840	0.716
rs2107595	LAS	*HDAC9*	A/G	intergenic variant	0.188	0.248
rs880315	AIS	*CASZ1*	C/T	intron variant	0.314	0.538
rs9515201	SVS	*COL4A2*	A/C	intron variant	0.321	0.593

AIS, Any ischemic stroke; ATXN2, ataxin 2; BNC2, basonuclin zinc finger protein 2; CASZ1, castor zinc finger 1; COL4A2, collagen type IV alpha 2 chain; EA, effect allele; EAF, effect allele frequency; H-W, Hardy–Weinberg equilibrium; HDAC9, histone deacetylase 9; IS, ischemic stroke; LAS, large artery stroke; OA, other allele; SVS, small vessel stroke; SH2B3, SH2B adaptor protein 3; SNP, single-nucleotide polymorphism; ZCCHC14, zinc finger CCHC-type containing 14.

**Table 3:** Allele frequency differences in IS-risk SNPs in AD.

SNP Allele	OR (95% CI)		*p* Value
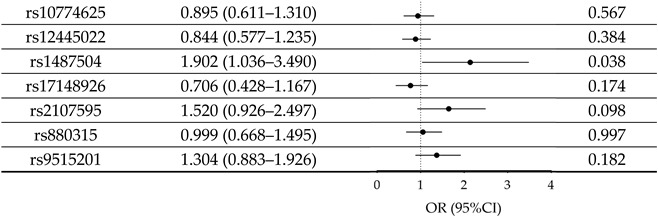			

AD, Alzheimer’s disease; CI: confidence interval; IS, ischemic stroke; OR, odds ratio; SNP, single-nucleotide polymorphisms. Logistic regression model in SPSS was used with corrections for age, gender, education year, and *apolipoprotein E* ε4 status.

**Table 4:** Allele frequency differences in rs1487504 in AD in with and without *ApoE* ε4 groups.

Allele	*ApoE* ε4 Status	OR	95% CI	*p* Value
rs1487504	*ApoE* ε4+	1.038	0.406–2.651	0.938
	*ApoE* ε4−	2.899	1.354–6.207	0.006 ^a^

AD, Alzheimer’s disease; ApoE, apolipoprotein E; CI: confidence interval; OR, odds ratio. ^a^: *p* < 0.00714

**Table 5:** GMDR analysis results of the interactions among IS-risk SNPs and *ApoE* ε4.

Model	Training Accuracy	Testing Accuracy	Sign Test (*p*)	Cross-Validation Consistency
*ApoE* ε4	0.697	0.6969	10 (0.0010)	10/10
rs1487504 *ApoE* ε4	0.7104	0.7043	10 (0.0010)	10/10
rs12445022 rs1487504 *ApoE* ε4	0.7265	0.6399	10 (0.0010)	4/10
rs1487504 rs17148926 rs9515201 *ApoE* ε4	0.758	0.651	9 (0.0107)	4/10
rs10774625 rs12445022 rs17148926 rs880315 *ApoE* ε4	0.802	0.6413	7 (0.1719)	8/10
rs10774625 rs12445022 rs17148926 rs2107595 rs880315 *ApoE* ε4	0.8543	0.5715	7 (0.1719)	5/10
rs10774625 rs12445022 rs17148926 rs2107595 rs880315 rs9515201 *ApoE* ε4	0.8977	0.5391	7 (0.1719)	10/10

AD, Alzheimer’s disease; ApoE, apolipoprotein E; IS, ischemic stroke; GMDR, generalized multifactor dimensionality reduction; SNP, single-nucleotide polymorphisms.

**Table 6:** Allele frequency differences in IS risk SNPs and AD CSF biomarkers in AD patients.

AD Biomarker	SNP Allele	BETA	t	*p* Value
CSF Aβ_42_ (pg/mL)	rs10774625	0.136	1.633	0.105
	rs12445022	0.020	0.241	0.810
	rs1487504	−0.197	−2.389	0.018 ^a^
	rs17148926	−0.161	−1.967	0.051
	rs2107595	−0.016	−0.191	0.849
	rs880315	0.238	2.924	0.004 ^b^
	rs9515201	−0.002	−0.029	0.977
CSF t-tau (pg/mL)	rs10774625	−0.014	−0.164	0.870
	rs12445022	0.129	1.541	0.126
	rs1487504	0.177	2.094	0.038 ^a^
	rs17148926	0.098	1.169	0.245
	rs2107595	0.160	1.873	0.063
	rs880315	0.079	0.924	0.358
	rs9515201	−0.143	−1.680	0.095
CSF p-tau_181_ (pg/mL)	rs10774625	−0.046	−0.521	0.603
	rs12445022	0.083	0.948	0.345
	rs1487504	0.038	0.432	0.666
	rs17148926	0.18	2.088	0.039 ^a^
	rs2107595	−0.016	−0.178	0.859
	rs880315	0.006	−0.066	0.947
	rs9515201	−0.059	−0.665	0.507

Aβ_42_, β-amyloid (1–42); AD, Alzheimer’s disease; BETA, regression coefficient; CSF, cerebrospinal fluid; IS, ischemic stroke; p-tau_181_, phosphorylated tau_181_; SNP, single-nucleotide polymorphisms; t, t-statistic; t-tau, total tau. Multiple linear regression model in SPSS was used with corrections for age, gender, education year, and *ApoE* ε4 status. ^a^: *p* < 0.05; ^b^: *p* < 0.00714.

**Table 7:** Association of IS-risk SNPs with AD neuroimaging biomarkers in AD patients.

	SNP Allele	BETA	t	*p* Value
Hippocampus (mm^3^)	rs10774625	−0.116	−1.421	0.158
	rs12445022	−0.041	−0.500	0.618
	rs1487504	−0.070	−0.859	0.392
	rs17148926	−0.074	−0.918	0.360
	rs2107595	−0.178	−2.180	0.031 ^a^
	rs880315	0.020	0.240	0.811
	rs9515201	0.072	0.882	0.379
Whole brain (mm^3^)	rs10774625	−0.149	−1.987	0.049 ^a^
	rs12445022	−0.087	−1.161	0.248
	rs1487504	0.010	0.129	0.898
	rs17148926	−0.116	−1.560	0.121
	rs2107595	−0.102	−1.333	0.185
	rs880315	0.093	1.224	0.223
	rs9515201	−0.045	−0.594	0.554
Entorhinal cortex (mm^3^)	rs10774625	−0.249	−2.929	0.004 ^b^
	rs12445022	−0.087	−1.008	0.315
	rs1487504	−0.081	−0.929	0.335
	rs17148926	−0.132	−1.542	0.126
	rs2107595	−0.196	−2.256	0.026 ^a^
	rs880315	0.093	1.062	0.290
	rs9515201	0.042	0.481	0.632
Mid-temporal lobe (mm^3^)	rs10774625	−0.035	−0.433	0.666
	rs12445022	−0.025	−0.317	0.752
	rs1487504	−0.076	−0.929	0.355
	rs17148926	0.016	0.197	0.844
	rs2107595	−0.001	−0.017	0.987
	rs880315	0.167	2.089	0.039 ^a^
	rs9515201	−0.155	−1.931	0.056

AD, Alzheimer’s disease; BETA, regression coefficient; IS, ischemic stroke; SNP, single-nucleotide polymorphisms; t, t-statistic t. Multiple linear regression model in SPSS was used with corrections for age, gender, education year, and *ApoE* ε4 status. ^a^: *p* < 0.05; ^b^: *p* < 0.00714.

**Table 8:** The possible connection between IS-risk SNPs and AD.

SNP	Gene	Potential Mechanisms That Connect IS and AD
rs1487504	*BNC2*	HF [29] and neuroinflammation [40]
rs10774625	*SH2B3/ATXN2*	Blood pressure variability [47] and decreased T1AM levels [48]
rs17148926	*LOC100505841*	White matter hyperintensities [42]
rs2107595	*HDAC9*	Neuroinflammation [51] and increased Aβ burden [53]
rs880315	*CASZ1*	T cell-associated inflammatory response [44]

Aβ, β-amyloid; AD, Alzheimer’s disease; ATXN2, ataxin 2; BNC2, basonuclin zinc finger protein 2; CASZ1, castor zinc finger 1; HDAC9, histone deacetylase 9; HF, heart failure; IS, ischemic stroke; SH2B3, SH2B adaptor protein 3; SNP, single-nucleotide polymorphisms; T1AM, TH derivative 3-iodothyronamine.

## Data Availability

The data utilized in this article were sourced from the Alzheimer’s Disease Neuroimaging Initiative (ADNI) database (http://adni.loni.usc.edu, accessed on 15 October 2024). The investigators associated with ADNI contributed to the design and implementation of the work and/or provided data; however, they did not engage in the analysis or the writing of this report. A comprehensive list of the ADNI investigators is available at the website http://adni.loni.usc.edu/wp-content/uploads/how_to_apply/ADNI_Acknowledgement_List.pdf. (accessed on 15 October 2024). The first author of this paper was granted administrative permissions to access the anonymized ADNI data since January 2021.

## Abbreviations

Aβ_42_	Amyloid-beta 1–42
AD	Alzheimer’s disease
ADNI	Alzheimer’s Disease Neuroimaging Initiative
AIS	Any ischemic stroke
ApoE	Apolipoprotein E
ATP5H	Adenosine triphosphate synthase subunit d, mitochondrial
ATXN2	Ataxin 2
BBB	Blood–brain barrier
BETA	Regression coefficient
BNC2	Basonuclin zinc finger protein 2
CASZ1	Castor zinc finger 1
CBF	Cerebral blood flow
CI	Confidence interval
COL4A2	Collagen type IV alpha 2 chain
CSF	Cerebrospinal fluid
EA	Effect allele
EAF	Effect allele frequency
ECM	Extracellular matrix
EPHA1	EPH receptor A1
eQTL	Expression quantitative trait loci
GMDR	Generalized multifactor dimensionality reduction
GWAS	Genome-wide association study
HDAC9	Histone deacetylase 9
HF	Heart failure
H-W	Hardy–Weinberg
ICH	Intracerebral hemorrhage
ICT1	Lysophosphatidic acid acyltransferase ICT1
IS	Ischemic stroke
KCTD2	Potassium channel tetramerization domain containing 2
LAS	Large artery stroke
LPS	Lipopolysaccharide
MCI	Mild cognitive impairment
MRI	Magnetic resonance imaging
MS4A4A	Membrane spanning 4-domains A4A
OA	Other allele
OR	Odds ratio
PET	Positron emission tomography
PPI	Protein–protein interactions
p-tau_181_	Phosphorylated tau 181
SH2B3	SH2B adaptor protein 3
SNP	Single-nucleotide polymorphism
SVS	Small vessel stroke
T1AM	Thyroid hormone derivative 3-iodothyronamine
Th	T helper
TH	Thyroid hormones
Treg	Regulatory T cell
TREM2	Triggering receptor expressed on myeloid cells 2
TSA	Trichostatin A
t-tau	Total tau
UBE2L3	Ubiquitin-conjugating enzyme E2 L3
WMH	White matter hyperintensities
ZCCHC14	Zinc finger CCHC-type containing 14
